# Lower transmissibility of SARS-CoV-2 among asymptomatic cases: evidence from contact tracing data in Oslo, Norway

**DOI:** 10.1186/s12916-022-02642-4

**Published:** 2022-11-08

**Authors:** Fredrik Methi, Elisabeth Henie Madslien

**Affiliations:** Norwegian Institute of Public Health, PO Box 222, Skøyen, N-0213 Oslo, Norway

**Keywords:** SARS-CoV-2, Asymptomatic, Transmission, Secondary attack rate, COVID-19

## Abstract

**Background:**

Asymptomatic COVID-19 cases have complicated the surveillance and tracking of the pandemic. Previous studies have estimated that 15–25% of all infectees remain asymptomatic.

**Methods:**

Based on contact tracing data from Oslo, Norway, we estimated transmission and susceptibility dynamics among symptomatic and asymptomatic cases and their contacts as identified by manual contact tracing between September 1, 2020, and September 1, 2021.

**Results:**

Among 27,473 indexes and 164,153 registered contacts, the secondary attack rate (SAR-14) was estimated to be 28% lower through asymptomatic exposure (13%) compared to symptomatic exposure (18%). Furthermore, those infected by asymptomatic cases were almost three times more likely to be asymptomatic compared to those infected by symptomatic cases.

**Conclusions:**

Symptomatic cases spread the virus to a greater extent than asymptomatic, and infectees are more likely to be asymptomatic if their assumed infector was asymptomatic.

**Supplementary Information:**

The online version contains supplementary material available at 10.1186/s12916-022-02642-4.

## Background

For more than 2 years, the COVID-19 pandemic has ravaged the world, and approximately one million research articles have been published concerning the pandemic [1]. Extensive focus has been put on the disease burden and ways to cope with the virus such as preventive measures and vaccination. However, less is known about those who get infected without developing symptoms. Asymptomatic cases have complicated the surveillance and tracking of the pandemic, as they are often not aware that they are infected and spread the virus unknowingly [2–4].

Systematic reviews have indicated that between 15 and 25% of all persons infected with SARS-CoV-2 have been asymptomatic [4, 5]. Yet, there are good reasons to believe that these numbers are grave underestimations, as many asymptomatic persons are never tested. So far, little is known about how these persons affect the spread of the virus. Previous meta-studies have found that asymptomatic cases have a relative transmissibility of 3–4 times less than symptomatic carriers [6, 7]. A caveat with these previous studies is that they mainly rely on follow-up cohort studies with few observations. The low number of observations makes data fragile to small adjustments [8]. A missing piece in the literature is utilising big data to see whether these findings hold for a larger share of the population and whether asymptomatic infectors are more likely to cause asymptomatic cases [7].

In this study, we utilised 1 year of contact tracing data from Oslo, Norway, to examine whether asymptomatic cases were less likely to spread the virus than symptomatic cases and whether cases were more likely to remain asymptomatic if their assumed infector was asymptomatic.

## Methods

### Study design

To estimate the impacts of asymptomatic carriers, we utilised population-wide longitudinal registry data from Norway. The Beredt-C19 register is an emergency preparedness register established as part of the legally mandated responsibilities of the Norwegian Institute of Public Health (NIPH) during epidemics [9]. The Beredt-C19 register compiles daily updated individual-level data from several registers. We started out using contact tracing data from Oslo municipality (PasInfo). This dataset included information on all SARS-CoV-2-positive cases registered in Oslo, their symptoms, date of testing, and information on their close contacts. We combined this data with register data on all registered reverse transcription polymerase chain reaction (PCR) tests and rapid antigen tests conducted in Norway from the Norwegian Surveillance System for Communicable Diseases Laboratory Database (MSIS-Lab) to retrieve tests for the close contacts. We also included data on vaccination from the Norwegian Immunisation Registry (SYSVAK). Finally, we included the National Population Register (FREG) to retrieve the characteristics of all persons included, such as age, sex, and country of birth. Ethical approval was obtained by the Regional Committees for Medical Research Ethics South East Norway (December 8th 2021, #400038).

### Study sample

Our target population included all residents in Oslo, Norway, who tested positive for SARS-CoV-2 between September 1, 2020, and September 1, 2021, and who were registered in the digital municipal contact tracing system (PasInfo) (*n* = 44,197). Manual contact tracing, isolation of positive cases, and quarantining close contacts started quickly as the epidemic hit Norway. Oslo started registering index cases from March 2020 and close contacts from May 2020. Testing was limited until the summer of 2020, with only the elderly, persons at risk, and health personnel being prioritised for testing [10]. From the summer of 2020 until the fall of 2021, testing was free and encouraged, and contact tracing was constantly operative. From September 27, 2021, contact tracers stopped registering close contacts outside the household [11]. We therefore limited the study period to September 1, 2020–September 1, 2021, to include a full year of operative contact tracing. Sensitivity analyses with all available data are included in appendix.

**Fig. 1:**
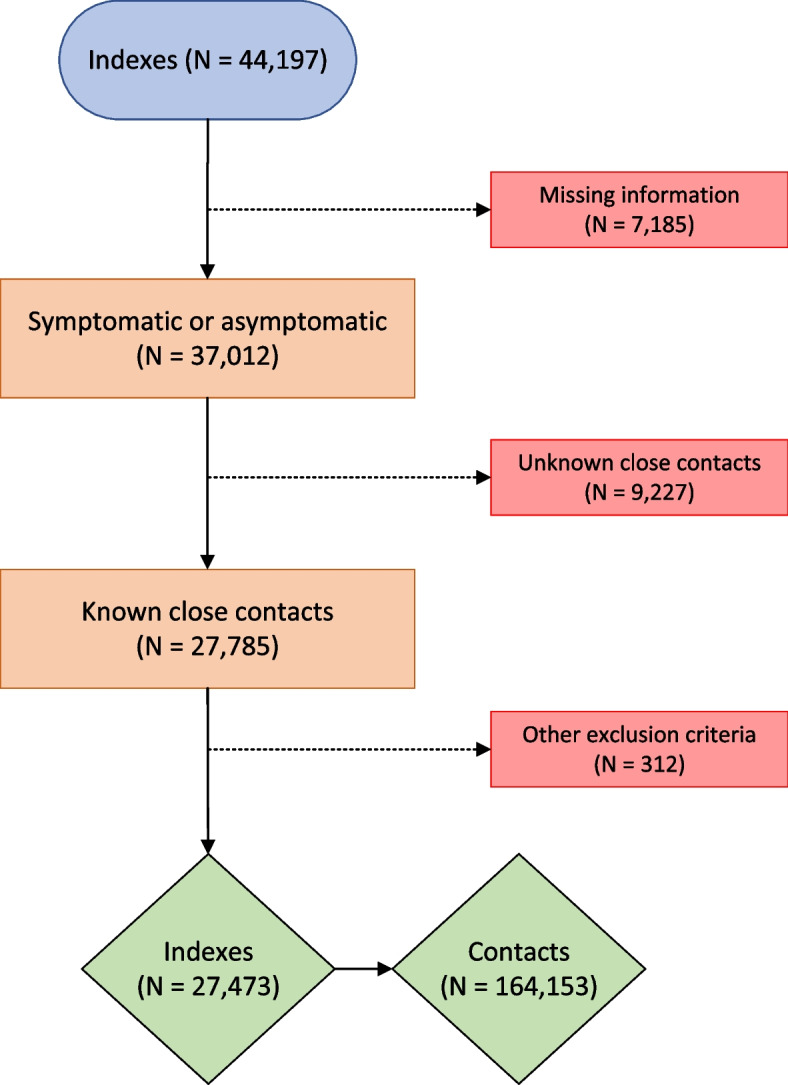
Flowchart of included index persons and close contacts. “Other exclusion criteria” includes index persons with more than 100 close contacts, index cases where all close contacts were missing in the population registry, or index cases where all close contacts had previously tested positive in the last 6 months

Of all 44,197 index persons registered in Oslo between September 1, 2020, and September 1, 2021, we excluded persons where information on symptoms was missing (*n* = 7185) and/or those without information on close contacts (*n* = 9227). Reasons for missing information on symptoms and/or close contacts may be that (1) not all persons had close contacts, some might have been isolated already prior to a positive test; (2) some refuse to provide information on close contacts; or (3) there was not enough capacity to perform contact tracing. We also excluded index cases registered with more than 100 close contacts (*n* = 25), index cases where all close contacts had previously tested positive within 6 months prior to index testing positive (*n* = 153), and indexes where none of the close contacts was available in the National Population Registry (*n* = 134). This left us with a total of 27,473 index cases and 164,153 close contacts (Fig. 1).

### Definitions

#### Index cases

All SARS-CoV-2 positive cases registered in the contact tracing system between September 1, 2020, and September 1, 2021, were defined as index cases. SARS-CoV-2 was either detected by PCR tests or rapid antigen tests (lateral flow) from samples (nasal and oropharyngeal swabs) taken by health personnel at official test stations. Of the 27,473 index cases, 26,766 (97.4%) were detected through PCR, 504 (1.9%) through both PCR and rapid antigen tests, and 199 (0.7%) through rapid antigen testing only. Only 4 cases (0.0%) had missing information on the method of testing. None of the indexes included in our data had previously tested positive for SARS-CoV-2.

#### Close contacts

Close contacts were identified through personal interviews with the index cases. The number of close contacts was self-reported by each index person when interviewed by the contact tracer. The definition of close contact has been constant throughout the pandemic [12]. A person was defined as a close contact if he or she had been in contact with a SARS-CoV-2-positive case less than 48 h before that person developed symptoms or tested positive. Contact was defined as having been closer than 2 m for more than 15 min, physical contact, or contact with secretions.

#### Asymptomatic/symptomatic cases

Information on COVID-19-related symptoms was registered by contact tracers employed by the municipality of Oslo. Information was collected through telephone interviews with each index case, usually within 2 days of a positive test. For most cases, symptoms were only collected at a single time point. Using this information, we classified people into two mutually exclusive groups: asymptomatic and symptomatic. A person was defined as asymptomatic if contact tracers registered the person as asymptomatic or with no COVID-19-related symptoms. Conversely, a person was classified as symptomatic if the person was registered with any COVID19-related symptoms. In a minor proportion of the cases, it was not entirely clear whether a person should be defined as asymptomatic or symptomatic. These cases were defined as follows: (a) “Person is asymptomatic now but had symptoms previously” was defined as symptomatic; (b) “Person is probably asymptomatic but suffers from dementia” was excluded; (c) “Person claims to be asymptomatic but sounds symptomatic” was excluded; and (d) “Person is asymptomatic with minor symptoms” was classified as asymptomatic. Since the majority of symptoms were reported within 2 days of positive tests, we could not differentiate between asymptomatic and presymptomatic cases (cases being asymptomatic at the time of reporting but developing symptoms later in the course of the disease).

## Statistical analyses

We first present descriptive statistics on the asymptomatic and symptomatic index cases.

Second, in line with previous literature, we defined secondary attack rates (SAR) as the per cent of the close contacts registered with a positive test, either PCR or antigen rapid test taken at official test stations, within 14 days after the index person tested positive [13–15]. The regime for testing, isolation, contact tracing, and quarantine has been changing over time. However, throughout the whole study period, close contacts have been encouraged to get tested (either by PCR or antigen rapid tests), but it has not been mandatory. As testing is a prerequisite for testing positive, we also ran sensitivity analyses including only close contacts registered with at least one (positive or negative) test within 14 days of index testing positive.

Finally, we estimated whether contacts infected by asymptomatic cases were more likely to stay asymptomatic compared to those infected by symptomatic cases. We excluded all contacts that had more than one index case where at least one of these was symptomatic and at least one was asymptomatic. If the contact had more than one index case and all of these were either symptomatic or asymptomatic, we included the contact in the analysis.

For both estimations, we relied on multiple logistic regression. We included personal characteristics such as sex (male/female), age (in 10-year intervals: 0–9, 10–19, ..., 60–69, 70+), country of birth (Norway/abroad), and vaccination status (1, 2, or 3 doses) for both the index person and the close contact, as well as calendar month to adjust for possible time trends (e.g. see Additional file 1: Supplementary Fig. 1 and Additional file 1: Supplementary Fig. 2) and the severeness of dominating mutations. Standard errors were clustered on the index case. All analyses were run in STATA SE v.16.

## Results

### Descriptive statistics

In Oslo, 27,473 positive COVID-19 cases (49.4% male and 50.6% female) with known close contacts were registered between September 1, 2020, and September 1, 2021, in the municipality’s contact tracing system (Table 1). Three thousand seven hundred sixty-five cases (13.7%) were defined as asymptomatic. Asymptomatic cases were on average younger, more often men, and had fewer vaccine doses than symptomatic cases at the time of positive test (Table 1). The share of asymptomatic indexes by each age is shown in Additional file 1: Supplementary Fig. 3.

**Table 1:** Descriptive statistics

	Asymptomatic				Symptomatic			
	Mean	Min	Max	95% CI	Mean	Min	Max	95% CI
Age	24.1	0	92	23.5–24.7	31.2	0	105	31.0–31.4
Male	0.54	0	1	0.53–0.55	0.49	0	1	0.48–0.49
Norwegian	0.62	0	1	0.61–0.64	0.63	0	1	0.62–0.63
Vaccines	0.07	0	3	0.06–0.08	0.12	0	3	0.11–0.12
Contacts	5.84	1	91	5.53–6.15	6.00	1	97	5.88–6.11

*Legend*: Table shows the descriptive statistics of asymptomatic and symptomatic index persons at the time of positive test

### Secondary attack rates

**Fig. 2:**
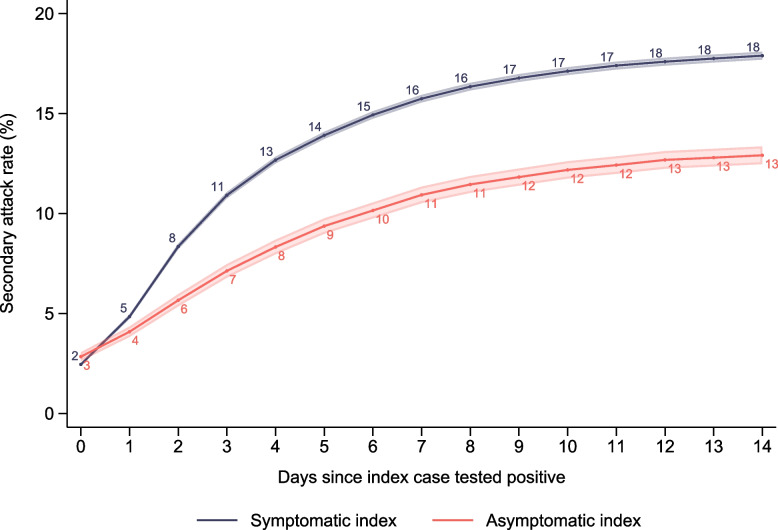
Probability of testing positive. Figure shows the share of close contacts testing positive within 14 days after index case tested positive. Shaded areas show the 95% confidence interval. Note that this is not conditional on being tested—all close contacts are included, tested or not. Additional file 1: Supplementary Fig. 4 presents the same figure only including those with at least one registered test within the 14-day time frame. And Additional file 1: Supplementary Fig. 5 presents the same figure stratified by household contacts and other contacts

Within 14 days of index cases testing positive, 78% of the 164,153 registered close contacts were tested (Additional file 1: Supplementary Fig. 4) and 17% tested positive (Fig. 2). When index case was registered as symptomatic, 18% [95% CI=18%18%] of the close contacts tested positive within 2 weeks (Fig. 2). On the other hand, when index case was registered as asymptomatic, the secondary attack rate was significantly lower at 13% [95% CI=12–13%] (Fig. 2), resulting in 28% lower relative risk for testing positive if a person was defined as a close contact to an asymptomatic index compared to a symptomatic index. The discrepancy remained after including only close contacts tested within 14 days (Additional file 1: Supplementary Fig. 5), and when distinguishing between household members and other close contacts (Additional file 1: Supplementary Fig. 6). We also estimated the SAR-14 for different age groups based on the age of the index case. For all age groups, except for the youngest (ages 0–9 years), the estimated SAR-14 was higher for symptomatic indexes than asymptomatic indexes (Additional file 1: Supplementary Fig. 7). Differences were not significant for ages 40–49 and 70+.

Using logistic regression we adjusted for age, sex, country of birth, and number of vaccine doses for the index case. In a separate model, we adjusted for the same confounders for both the index case and the close contact. Neither model changed the results. The odds of testing positive were ∼30% lower for persons if their assumed index person was asymptomatic rather than symptomatic (Table 2).

**Table 2:** Odds ratio of being infected by asymptomatic persons

	Crude	+ Index controls	+ Case controls
Asymptomatic	0.68	0.74	0.73
	(0.63–0.74)	(0.68–0.80)	(0.67–0.79)
Symptomatic	Ref	Ref	Ref
	(.)	(.)	(.)
Number of indexes	27,473	27,134	26,772
Number of close contacts	164,153	163,190	161,298

*Legend*: Table shows the odds ratio of testing positive within 14 days if the case was defined as a close contact to an asymptomatic case compared to a symptomatic case. Crude shows the unadjusted ratio; + Index controls include confounders for the index case (age, sex, country of birth, and number of vaccine doses); and + Case controls additionally adjust for these confounders for the close contact. Note that this is not conditional on being tested. For full regression models and conditionality on being tested see Additional file 1: Supplementary Table 1

Throughout the pandemic close contacts had been encouraged to test themselves, but not forced to do so. We therefore ran sub-analyses including only close contacts registered with at least one PCR test (negative or positive) within 14 days (Additional file 1: Supplementary Table 1). This did not alter the results (odds ratio (OR) = 0.70). We also ran separate analyses comparing only persons identified household contacts and another analysis only including those defined as other types of contacts, and the effect remained (0.67 vs. 0.67). Making use of all available contact tracing data (i.e. not limiting the study period to 1 year) also provided similar results (Additional file 1: Supplementary Table 2). Similarly, including indexes with missing information on symptoms (*n* = 7185) either as symptomatic (OR = 0.71) or asymptomatic (OR = 0.70) did not substantially alter the results.

### Asymptomatic vs symptomatic

Furthermore, we examined whether infectees were more likely to remain asymptomatic if their assumed infector was registered as asymptomatic. This analysis included all close contacts who tested positive within 14 days after their index case tested positive. We excluded close contacts testing positive but where information on symptoms was missing. Likewise, we excluded close contacts linked to more than one index case and these indexes were not both asymptomatic or both symptomatic. This left us with a total of 11,192 positive close contacts and 7786 index cases.

In total, 16% of all close contacts who tested positive within 14 days were registered as asymptomatic. This share was drastically higher if the index case was asymptomatic (32%) compared to if the index case was symptomatic (15%). Hence, infectees had more than twice the probability of remaining asymptomatic if their assumed infector was asymptomatic, not adjusted for confounding factors. As shown in both Additional file 1: Supplementary Fig. 3 and Additional file 1: Supplementary Fig. 8, children aged 0–9 years old were more likely to be asymptomatic.

In Table 3, we ran several logistic regression models with the likelihood of being asymptomatic as the outcome variable. We used the same confounders as in the previous SAR model. This time, the first model being a crude model without any confounders, the second model adjusting for characteristics of the secondary case, and the third model adjusting for both characteristics of the secondary case and the index person. Full regression models are available in Additional file 1: Supplementary Table 3. All three models estimated that infectees were two to three times more likely to remain asymptomatic (OR being 2.67, 2.69, and 2.72, respectively) if their assumed infector was registered as asymptomatic (Table 3).

**Table 3:** Odds ratio of being asymptomatic if the index is asymptomatic compared to symptomatic

	Crude	+ Case controls	+ Index controls
Asymptomatic	2.67	2.69	2.72
	(2.28–3.12)	(2.27–3.19)	(2.27–3.26)
Symptomatic	Ref	Ref	Ref
	(.)	(.)	(.)
Number of indexes	7786	7715	7689
Number of cases	11,192	11,105	11,066

*Legend*: Table shows the odds ratio of being asymptomatic if your assumed infector was asymptomatic compared to symptomatic. Crude shows the unadjusted ratio; + Case controls include confounders for the secondary person (age, sex country of birth and number of vaccine doses); and + Index controls additionally adjust for these confounders for the index person. Note that this is not conditional on being tested. For full regression models and conditionality on being tested see Additional file 1: Supplementary Table 3.

## Discussion

In this study of all 27,473 persons testing positive for SARS-CoV-2 from September 1, 2020, to September 1, 2021, in Oslo, registered with information on symptoms and close contacts, we found that asymptomatic cases were almost 30% less transmissible than symptomatic cases. Examining the 11,192 secondary cases we also found that infectees were almost three times more likely to remain asymptomatic if their assumed infector was asymptomatic compared to symptomatic.

### Principal findings

The findings of both analyses were in line with our hypotheses and previous research. There are several reasons why asymptomatic cases may be less transmissible than symptomatic. First, the lack of coughing, sneezing, and other respiratory symptoms may reduce the spread of respiratory droplets and make asymptomatic cases less transmissible [2]. Differences in viral load and viral shedding between the two groups may also partly explain the differences in transmissibility [4], but so far, the literature on the relationship between viral load and disease severity is inconclusive [16]. Finally, there may be differences in the behavioural patterns of symptomatic and asymptomatic persons. Asymptomatic cases may perceive themselves, and being perceived by others, as not being infectious, which could lead to infection chains not detectable through contact tracing [17]. Symptomatic persons might to a larger extent have close contacts they are not possible to be isolated from (e.g. mother and child). Whereas asymptomatic persons (not knowing they are positive) may have more close contact with less intimacy. On the other hand, it might also be the case that symptomatic persons are already aware that they are ill and take precautionary measures, whereas asymptomatic individuals do not take any measures. We tried to examine this as best as possible by distinguishing between household contacts and other close contacts (most often being students, colleagues, and friends) (Additional file 1: Supplementary Fig. 6). This did not alter the results. Moreover, we found no differences in the number of registered close contacts between asymptomatic and symptomatic cases.

We also found that most asymptomatic persons were young children. At the start of the pandemic, children were rarely infected, and if they were infected, they had a lower transmissibility than adults [18]. Previous studies have revealed that little transmission has taken place in schools during the pandemic [18, 19]. During our study period schools mostly used precautionary measures such as quarantine and homeschooling. At the end of the study period, strict testing criteria replaced quarantine and homeschooling, which could result in more asymptomatic cases being detected. However, as this was not widely implemented before the autumn of 2021, it is not redeemed likely to explain why we see more young persons being asymptomatic. The situation of exposure may differ between young children and adults.

However, we found no differences in the share being asymptomatic based on the type of close contact for the different age groups (Additional file 1: Supplementary Table 4). The share of asymptomatic cases was equally distributed among secondary cases registered as household members and other types of close contacts.

Furthermore, we found differences in transmission dynamics between asymptomatic and symptomatic for all age groups except the youngest (0–9 years). We have no evidence as to why there were no differences for the youngest age group, but reasons might be that it can be hard to distinguish between symptomatic and asymptomatic for the youngest children. For toddlers and young children, contact tracers have interviewed their parents and not the case itself, which in turn might complicate the accuracy of reported symptoms.

### Related studies

Our findings are in line with previous research. Whereas previous studies found that asymptomatic persons had a relative transmissibility three to four times lower than symptomatic persons [6, 7], we found this to be 0.3–0.4 times lower. One reason for this discrepancy might be that asymptomatic persons more seldom test themselves. In follow-up studies, all persons get tested regularly, whereas in registry studies researchers are dependent on persons physically visiting testing centres to get tested. This might be more common for cases that are symptomatic compared to asymptomatic. We also found that the relative transmissibility was evident for all age groups except for the youngest. Evidence from other studies suggests that transmission dynamics in households are relatively similar across age groups [20].

Similarly, we found that the average age of asymptomatic cases (24.1) was lower than that of symptomatic cases (31.2). Similar results were found in a meta-study analysing over 350 studies, where the authors found that the share of elderly being asymptomatic was significantly lower than children, with 19.7% compared to 46.7% [21].

As previously hypothesised, we found that secondary cases were two to three times more likely to be asymptomatic if their assumed index case was asymptomatic. To the authors’ knowledge, this is the first study to assess whether asymptomatic indexes are more likely to cause asymptomatic secondary cases.

### Strengths and weaknesses

A major strength with this register study is that we could include all persons registered with positive or negative PCR or rapid antigen tests in Oslo throughout the whole study period. Furthermore, health registry data are mandated in Norway and are generally of very high validity and reliability. The large number of observations provides more statistical power, less uncertainty, and more generalisability to the findings. Combining registry data with qualitative contact tracing data gathered through interviews with trained personnel and infectees provides an invaluable dynamic to the study. During the 1-year study period, Norway, and Oslo, had eminent test and contact tracing capacity, meaning it is reasonable to assume that a large share of the real number of index cases and their close contacts were identified and included in this study.

There are also some potential limitations with this study. Information on symptoms was gathered through telephone calls to the infectees and was therefore self-reported. Men were more often reported to be asymptomatic than women in our data. We could not be certain whether men actually were more often asymptomatic, or whether this was an example of men having a higher threshold of reporting symptoms [22]. Differences between various contact tracers in what they regarded as asymptomatic may also have affected the data, but probably only to a minor extent as they all were professionally trained. Moreover, the national strategy on testing, isolation, contact tracing, and quarantining changed several times during the pandemic. Particularly, changes in recommendations on testing for close contacts could have affected the results in this study. Nonetheless, it is reassuring that results seemed to be constant across the whole study period (Additional file 1: Supplementary Fig. 2).

A second limitation is that we were not able to distinguish between asymptomatic and presymptomatic persons. In theory, a person is asymptomatic if the person tests positive but do not develop symptoms throughout the course of the disease. A presymptomatic person, on the other hand, is a person who develops symptoms at a later date [23]. As contact tracers usually contacted positive persons once, different timing in the course of the disease may have resulted in presymptomatic being reported as asymptomatic. However, presymptomatic persons were still below the detection of the healthcare system and may have continued spreading the virus.

Moreover, persons with false-positive tests may have been classified as asymptomatic in registry data. Although PCR tests are considered to be highly accurate and with high sensitivity and specificity, false-negative and false-positive tests still occur [24]. In diseases with low prevalence, small errors in specificity may result in a large share of false positives, also known as the false-positive paradox [25, 26]. In our data, only 13% of all SARS-CoV-2-positive cases were registered as asymptomatic, which was lower than the previous estimates of 15–25% [4, 5], suggesting that the share of asymptomatic persons in our population was underestimated rather than overestimated.

Finally, we did not have information on Ct values or viral load in each test, and we were therefore not able to tell whether differences in viral load between asymptomatic and symptomatic cases may explain the lower transmissibility.

### Future research

For future research, it would be interesting to examine whether cycle threshold (Ct) values and viral load varies between symptomatic and asymptomatic cases; whether having an asymptomatic course of the disease lowers the risk of developing post-acute sequelae known as “long-COVID”; and whether there are differences in the probability of remaining asymptomatic based on the different lineages of the virus, number of vaccine doses, or types of vaccine. Moreover, further investigating how these transmission and susceptibility dynamics differ between different age groups may be important for future measures regarding school closures and measures drastically impacting children’s everyday life.

## Conclusions

We found transmissibility to be approximately 30% lower among asymptomatic persons (13%) than symptomatic persons (18%). This remained after adjusting for individual characteristics both of the infector and the potentially infected person and for time trends. Moreover, we found evidence that infectees were up to three times more likely to remain asymptomatic if their assumed infectors were asymptomatic compared to symptomatic.

## Supplementary Information

**Additional file 1: Supplementary Fig. 1.** Share of asymptomatic cases by date. **Supplementary Fig. 2.** SAR-14 by calendar month. **Supplementary Fig. 3.** Share of indexes being asymptomatic by age. **Supplementary Fig. 4.** Share of close contacts tested within 14 days. **Supplementary Fig. 5.** Probability of testing positive conditional on testing. **Supplementary Fig. 6.** Probability of testing positive by type of close contact. **Supplementary Fig. 7.** SAR-14 by age group. **Supplementary Table 1.** Logistic regression. Probability on being infected. **Supplementary Table 2.** Logistic regression. Full sample. **Supplementary Table 3.** Logistic regression. Asymptomatic causation. **Supplementary Fig. 8.** Age combinations of asymptomatic transmission. **Supplementary Table 4.** Asymptomatic by type of close contact and age.

## Data Availability

The data that support the findings of this study are available from the Norwegian Institute of Public Health (Norwegian Immunization Registry and Norwegian Surveillance System for Communicable Diseases Laboratory Database), Norwegian Tax Administration (National Population Register) and Oslo municipality (PasInfo) but restriction apply to the availability of these data, which were used under licence for the current study, and so are not publicly available. Data are however available from the authors upon reasonable request and with permission of the Norwegian Directorate of Public Health, Norwegian Institute of Public Health, Norwegian Tax Administration, and Oslo municipality.

## Abbreviations

Beredt-C19: Norwegian Emergency Preparedness Register
COVID-19: Coronavirus disease 2019
Ct: Cycle threshold
FREG: National Population Register
MSIS-Lab: Norwegian Surveillance System for Communicable Disease Laboratory Database
NIPH: Norwegian Institute of Public Health
OR: Odds ratio
PasInfo: Contact tracing data from Oslo municipality
PCR: Polymerase chain reaction
SAR: Secondary attack rate
SARS-CoV-2: Severe acute respiratory syndrome coronavirus 2
SYSVAK: Norwegian Immunization Registry
