# Supplementary material for: Lower transmissibility of SARS-CoV-2 among asymptomatic cases: evidence from contact tracing data in Oslo, Norway
Source: BMC Med. 2022 Nov 8;20:427. doi: 10.1186/s12916-022-02642-4 (PMC9641677; doi:10.1186/s12916-022-02642-4)
Supplement: Supplementary file 1 — Additional file 1: Supplementary Fig. 1. Share of asymptomatic cases by date. Supplementary Fig. 2. SAR-14 by calendar month. Supplementary Fig. 3. Share of indexes being asymptomatic by age. Supplementary Fig. 4. Share of close contacts tested within 14 days. Supplementary Fig. 5. Probability of testing positive conditional on testing. Supplementary Fig. 6. Probability of testing positive by type of close contact. Supplementary Fig. 7. SAR-14 by age group. Supplementary Table 1. Logistic regression. Probability on being infected. Supplementary Table 2. Logistic regression. Full sample. Supplementary Table 3. Logistic regression. Asymptomatic causation. Supplementary Fig. 8. Age combinations of asymptomatic transmission. Supplementary Table 4. Asymptomatic by type of close contact and age. [file 12916_2022_2642_MOESM1_ESM.pdf]

Supplementary file for the paper

Lower transmissibility of SARS-CoV-2 among asymptomatic cases: Evidence from contact tracing data in Oslo, Norway.

By Methi & Madslien, 2022.

(Short titles)

**Supplementary Fig 1:** Share of asymptomatic cases by date.	p. 2
**Supplementary Fig 2:** SAR-14 by calendar month.	p. 3
**Supplementary Fig 3:** Share of indexes being asymptomatic by age.	p. 4
**Supplementary Fig 4:** Share of close contacts tested within 14 days.	p. 5
**Supplementary Fig 5:** Probability of testing positive conditional on testing.	p. 6
**Supplementary Fig 6:** Probability of testing positive by type of close contact.	p. 7
**Supplementary Fig 7:** SAR-14 by age group.	p. 8
**Supplementary Table 1:** Logistic regression. Probability of being infected.	p. 9
**Supplementary Table 2:** Logistic regression. Full sample.	p. 10
**Supplementary Table 3:** Logistic regression. Asymptomatic causation.	p. 11
**Supplementary Fig 8:** Age combinations of asymptomatic transmission.	p. 12
**Supplementary Table 4:** Asymptomatic by type of close contact and age.	p. 12

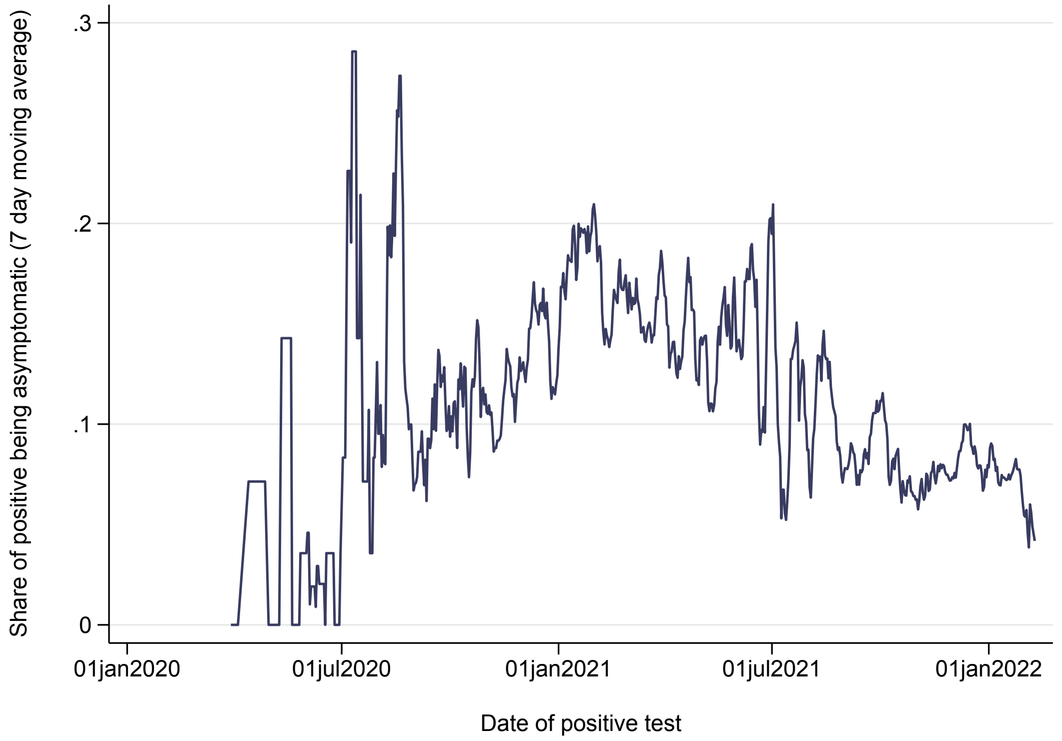

Note: Figure showing the share of asymptomatic among all positive SARS-CoV-2 tests in Oslo by given the given date. Shares are calculated as a 7-day moving average.

Supplementary Fig 1: Share of asymptomatic cases by date.

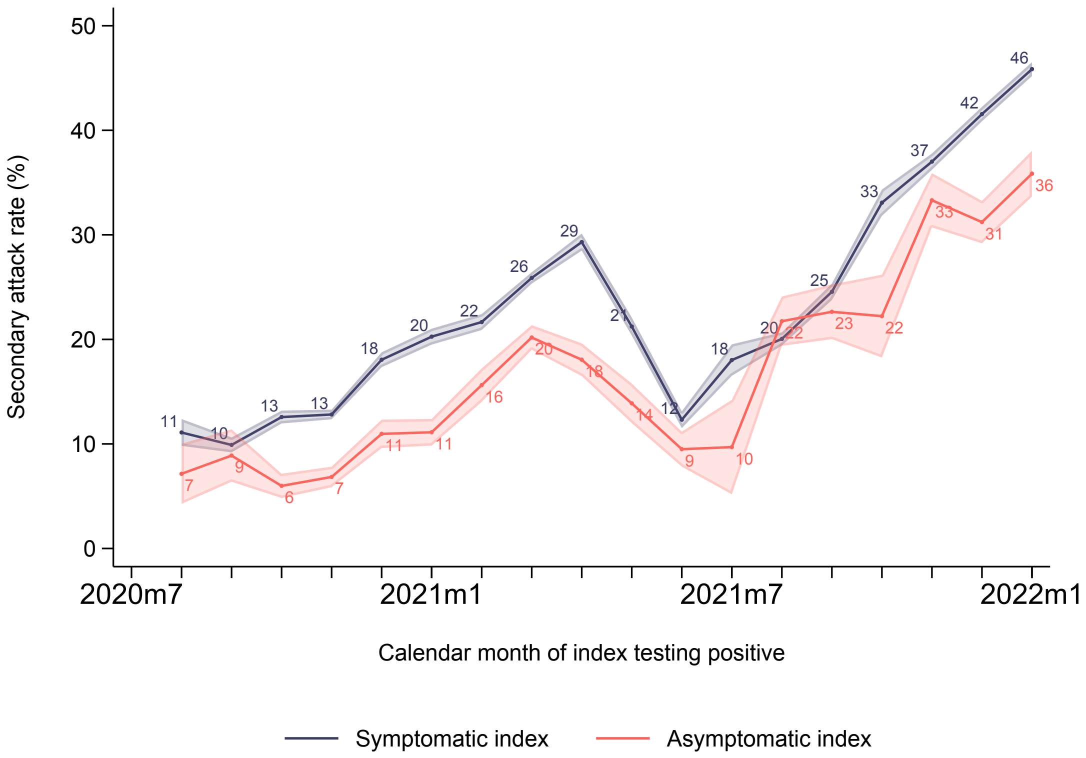

Note: Figure showing the monthly secondary attack rate (SAR-14) for symptomatic and asymptomatic indexes. The figure only includes months with at least 100 observations of symptomatic and asymptomatic indexes. Shaded areas show the 95% confidence interval.

Supplementary Fig 2: SAR-14 by calendar month.

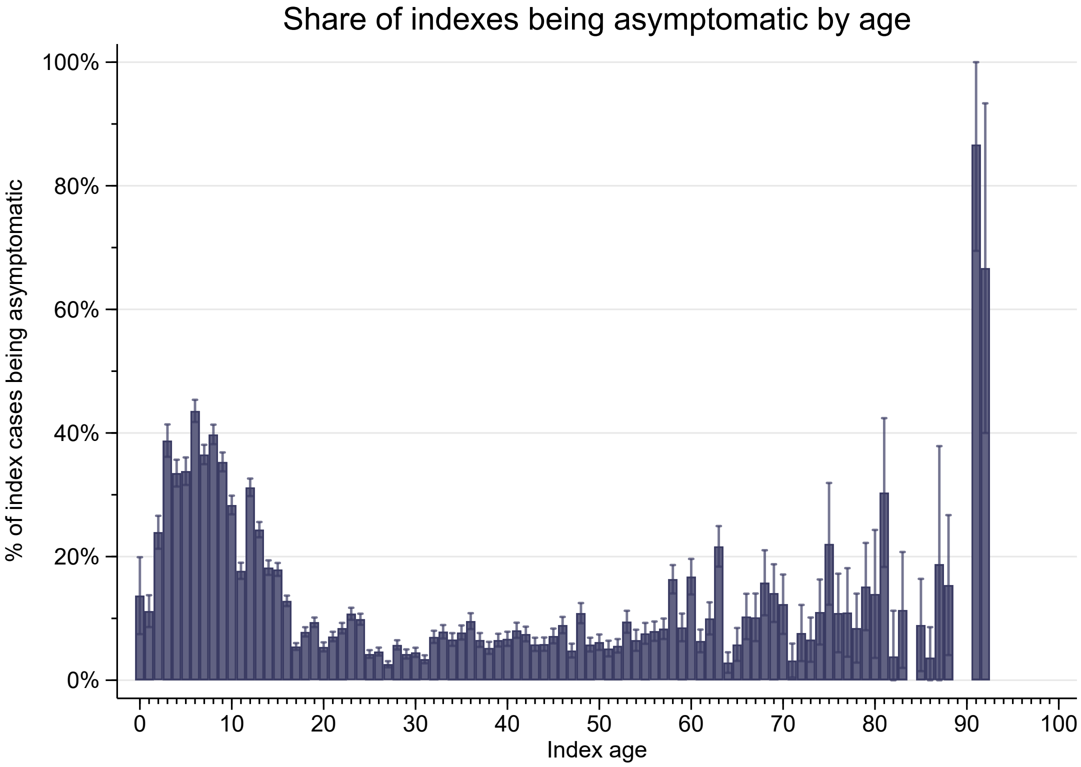

Note: Bar shows the percentage of indexes registered as asymptomatic by age. Spikes show the upper and lower 95% confidence intervals.

Supplementary Fig 3: Share of indexes being asymptomatic by age.

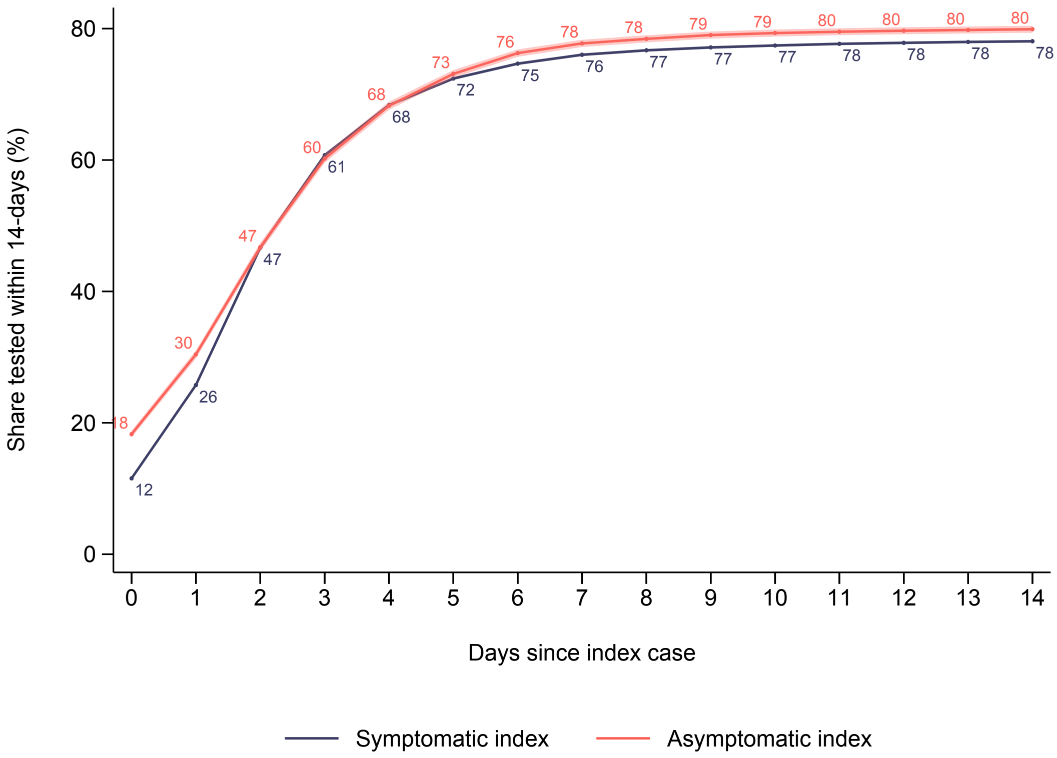

Note: Figure shows the share of close contacts that are registered with at least one (positive or negative) PCR-test within 14 days of index testing positive. Shaded areas show the 95% confidence interval.

Supplementary Fig 4: Share of close contacts tested within 14 days.

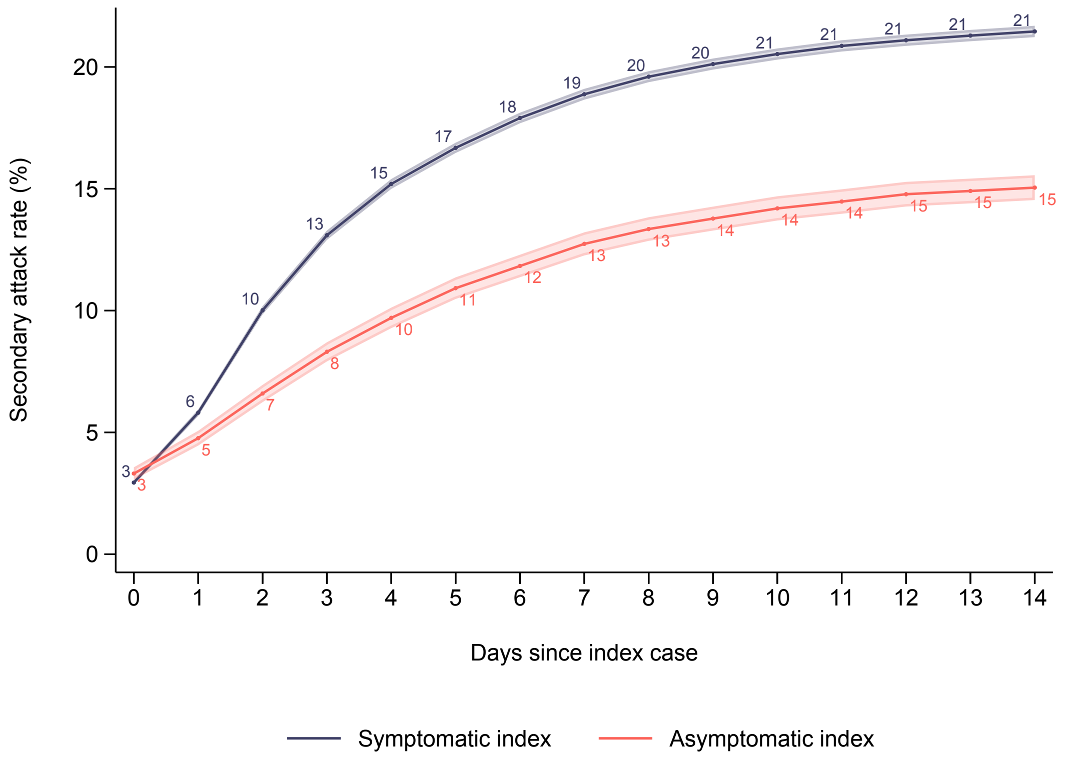

Note: Figure shows the share of close contacts testing positive within 14 days of index testing positive, and only includes close contacts registered with at least one test within these 14 days. Shaded areas show the 95% confidence interval.

Supplementary Fig 5: Probability of testing positive conditional on testing.

(a) Household members (b) Other close contacts

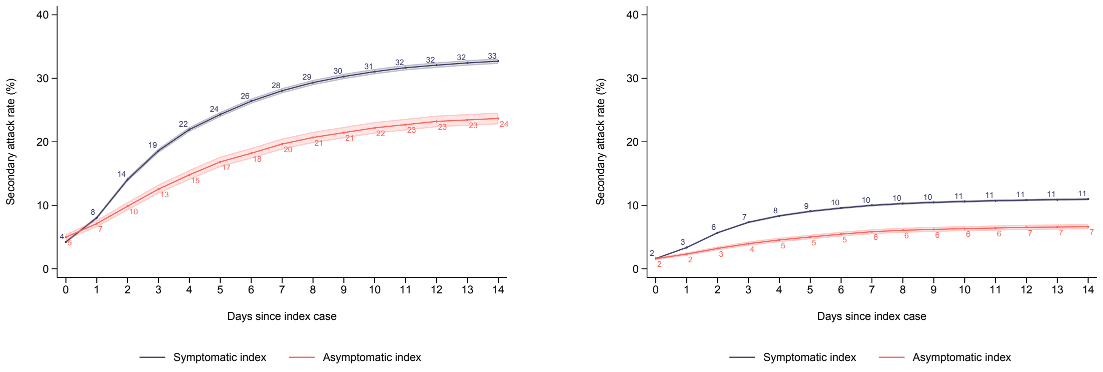

Note: Figure shows the share of close contacts testing positive within 14 days of index testing positive. Panel (a) only includes household members, and panel (b) include all other types of close contacts not being household members. Shaded areas show the 95% confidence interval.

Supplementary Fig 6: Probability of testing positive by type of close contact.

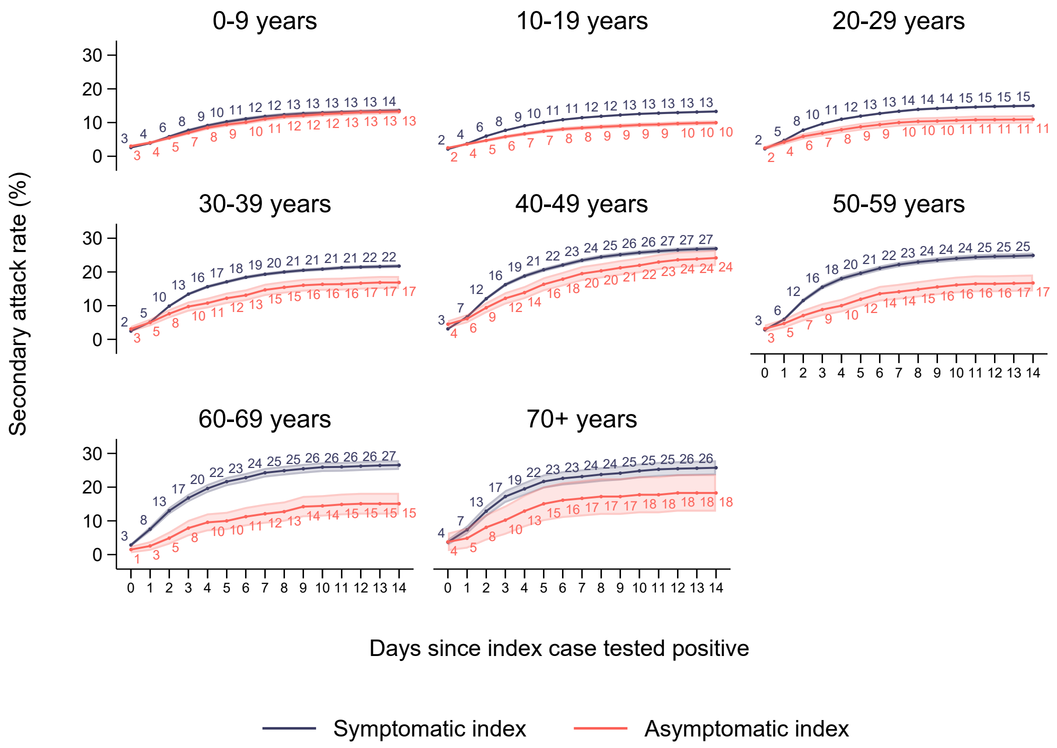

Note: Figure showing the monthly secondary attack rate (SAR-14) for symptomatic and asymptomatic indexes. The figure only includes months with at least 100 observations of symptomatic and asymptomatic indexes. Shaded areas show the 95% confidence interval.

Supplementary Fig 7: SAR-14 by age groups.

Supplementary Table 1: Logistic regression. Probability of being infected.

Model (1) shows the crude model with zero confounders. Model (2) adjusts for characteristics for the index case. Model (3) adjusts for characteristics for both the index and the close contact. Model (4) only includes the persons with at least one registered PCR-test within 14 days, adjusting for characteristics for both the index and the close contact. Model (5) only includes close contacts defined as household-members, adjusting for characteristics for both the index and the close contact. Model (6) only includes close contacts not defined as household-members, adjusting for characteristics for both the index and the close contact. Model 2-6 includes time fixed effects for calendar months.

	(1)<br>Crude	(2)<br>Index	(3)<br>Case	(4)<br>Tested	(5)<br>Household	(6)<br>Other
Asymptomatic	0.68***	0.74***	0.73***	0.70***	0.67***	0.67***
	(0.01)	(0.02)	(0.02)	(0.02)	(0.02)	(0.02)
INDEX						
10-19 years		0.88***	0.82***	0.85***	0.70***	1.02
		(0.02)	(0.02)	(0.02)	(0.03)	(0.05)
20-29 years		1.00	0.85***	0.89***	0.79***	1.23***
		(0.03)	(0.02)	(0.03)	(0.03)	(0.06)
30-39 years		1.41***	1.25***	1.27***	1.29***	1.66***
		(0.04)	(0.04)	(0.04)	(0.05)	(0.08)
40-49 years		1.82***	1.60***	1.61*	1.55	2.02***
		(0.05)	(0.05)	(0.05)	(0.06)	(0.10)
50-59 years		1.61***	1.39***	1.45***	1.35**	1.87***
		(0.05)	(0.05)	(0.05)	(0.06)	(0.10)
60-69 years		1.78***	1.52***	1.60***	1.24***	2.50***
		(0.08)	(0.07)	(0.08)	(0.08)	(0.16)
70+ years		1.85***	1.61***	1.69***	1.09	2.82***
		(0.11)	(0.10)	(0.11)	(0.11)	(0.24)
Male		1.15***	1.12***	1.13***	1.11***	1.10***
		(0.01)	(0.01)	(0.01)	(0.02)	(0.02)
Norwegian		0.64***	0.62***	0.61***	0.71***	0.73***
		(0.01)	(0.01)	(0.01)	(0.02)	(0.02)
1 dose		0.77***	0.98	1.02	1.03	0.91*
		(0.02)	(0.03)	(0.04)	(0.05)	(0.05)
2 doses		1.79***	1.77***	2.01***	1.24***	1.63***
		(0.03)	(0.03)	(0.04)	(0.02)	(0.08)
3 doses		0.90	1.71	1.45	1.55	1
		(0.08)	(0.08)	(0.11)	(0.06)	(.)
CASE						
10-19 years			1.20***	1.08***	1.11***	1.47***
			(0.03)	(0.03)	(0.03)	(0.06)
20-29 years			1.52***	1.29***	1.67***	1.96***
			(0.04)	(0.03)	(0.06)	(0.07)
30-39 years			1.38***	1.19***	1.88***	1.37***
			(0.03)	(0.03)	(0.07)	(0.05)
40-49 years			1.70***	1.49***	1.82***	1.49***
			(0.04)	(0.04)	(0.06)	(0.07)
50-59 years			1.46***	1.29***	1.66***	1.33***
			(0.04)	(0.04)	(0.07)	(0.06)
60-69 years			1.28***	1.19***	1.92***	1.41***
			(0.05)	(0.05)	(0.13)	(0.08)
70+ years			0.94	0.84***	3.31***	1.22***
			(0.05)	(0.04)	(0.35)	(0.08)
Male			1	1	1	1
			(.)	(.)	(.)	(.)
Norwegian			1	1	1	1
			(.)	(.)	(.)	(.)
1 dose			0.74***	0.77***	0.59***	0.76***
			(0.03)	(0.03)	(0.03)	(0.04)
2 doses			0.28***	0.42***	0.20***	0.22***
			(0.01)	(0.02)	(0.01)	(0.02)
3 doses			1.14	1.12	1	10.5*
			(1.28)	(1.30)	(.)	(14.87)
Time trends	No	Yes	Yes	Yes	Yes	Yes
Indexes	27,473	27,134	26,804	22,319	13,400	13,404
Cases	164,153	163,190	161,298	127,909	52,508	108,790

Odds ratios; Standard errors in parentheses
* $p < 0.05$, ** $p < 0.01$, *** $p < 0.001$

Supplementary Table 2: Logistic regression. Full sample.

Model (1) shows the crude model with zero confounders. Model (2) adjusts for characteristics for the index case. Model (3) adjusts for characteristics for both the index and the close contact. Model (4) only includes the persons with at least one registered PCR-test within 14 days, adjusting for characteristics for both the index and the close contact. Model (5) only includes close contacts defined as household-members, adjusting for characteristics for both the index and the close contact. Model (6) only includes close contacts not defined as household-members, adjusting for characteristics for both the index and the close contact. Model 2-6 includes time fixed effects for calendar months.

	(1)<br>Crude	(2)<br>Index	(3)<br>Case	(4)<br>Tested	(5)<br>Household	(6)<br>Other
Asymptomatic	0.62***	0.61***	0.62***	0.58***	0.62***	0.61***
	(0.01)	(0.01)	(0.01)	(0.01)	(0.01)	(0.02)
INDEX						
10-19 years		0.65***	0.65***	0.66***	0.62***	0.94
		(0.01)	(0.01)	(0.01)	(0.01)	(0.04)
20-29 years		0.53***	0.53***	0.54***	0.63***	1.04
		(0.01)	(0.01)	(0.01)	(0.02)	(0.04)
30-39 years		0.77***	0.77***	0.79***	0.89***	1.38***
		(0.01)	(0.02)	(0.02)	(0.02)	(0.06)
40-49 years		0.93***	0.94***	0.96*	1.03	1.66***
		(0.02)	(0.02)	(0.02)	(0.03)	(0.07)
50-59 years		0.77***	0.80***	0.83***	0.93**	1.53***
		(0.02)	(0.02)	(0.02)	(0.03)	(0.07)
60-69 years		0.82***	0.85***	0.88***	0.88***	2.03***
		(0.03)	(0.03)	(0.03)	(0.04)	(0.12)
70+ years		0.84***	0.93	0.96	0.93	2.17***
		(0.04)	(0.04)	(0.05)	(0.06)	(0.17)
Male		1.18***	1.16***	1.17***	1.15***	1.13***
		(0.01)	(0.01)	(0.01)	(0.01)	(0.02)
Norwegian		0.68***	0.68***	0.64***	0.81***	0.74***
		(0.01)	(0.01)	(0.01)	(0.01)	(0.01)
1 dose		1.14***	1.12***	1.22***	1.01	1.00
		(0.02)	(0.02)	(0.03)	(0.02)	(0.04)
2 doses		1.79***	1.77***	2.01***	1.24***	1.63***
		(0.03)	(0.03)	(0.04)	(0.02)	(0.08)
3+ doses		2.07***	2.01***	2.16***	1.34***	2.39***
		(0.08)	(0.08)	(0.11)	(0.06)	(0.38)
CASE						
10-19 years			1.05***	0.92***	1.10***	1.43***
			(0.02)	(0.02)	(0.02)	(0.05)
20-29 years			1.16***	1.01	1.39***	1.84***
			(0.02)	(0.02)	(0.03)	(0.06)
30-39 years			1.34***	1.13***	1.88***	1.33***
			(0.02)	(0.02)	(0.04)	(0.05)
40-49 years			1.42***	1.25***	1.55***	1.54***
			(0.03)	(0.03)	(0.04)	(0.06)
50-59 years			1.10***	0.99	1.27***	1.37***
			(0.02)	(0.03)	(0.03)	(0.06)
60-69 years			0.97	0.89***	1.35***	1.38***
			(0.03)	(0.03)	(0.06)	(0.07)
70+ years			0.67***	0.55***	1.68***	1.01
			(0.03)	(0.02)	(0.10)	(0.06)
Male			1	1	1	1
			(.)	(.)	(.)	(.)
Norwegian			1	1	1	1
			(.)	(.)	(.)	(.)
1 dose			0.93***	0.98	0.70***	0.82***
			(0.02)	(0.02)	(0.02)	(0.04)
2 doses			1.01	1.35***	0.63***	0.63***
			(0.02)	(0.03)	(0.01)	(0.03)
3+ doses			1.13***	1.62***	0.60***	1.25*
			(0.04)	(0.06)	(0.02)	(0.15)
Time trends	No	Yes	Yes	Yes	Yes	Yes
Indexes	64,491	64,018	64,017	41,609	47,022	16,995
Cases	245,813	244,647	244,641	166,266	123,991	120,650

Odds ratios; Standard errors in parentheses
* $p < 0.05$, ** $p < 0.01$, *** $p < 0.001$

Supplementary Table 3: Logistic regression. Probability of being asymptomatic if your assumed infector was asymptomatic.

Model (1) shows the crude model with zero confounders. Model (2) adjusts for characteristics for the secondary case. Model (3) adjusts for characteristics for both the close contact and the index. Model 2 and 3 includes time fixed effects for calendar months.

	(1)<br>Crude	(2)<br>Case	(3)<br>Index
Asymptomatic index	2.67***	2.69***	2.72***
	(0.21)	(0.23)	(0.25)
CASE			
10-19 years		0.39***	0.40***
		(0.03)	(0.03)
20-29 years		0.19***	0.18***
		(0.02)	(0.02)
30-39 years		0.17***	0.16***
		(0.02)	(0.02)
40-49 years		0.18***	0.18***
		(0.02)	(0.02)
50-59 years		0.21***	0.20***
		(0.02)	(0.02)
60-69 years		0.24***	0.23***
		(0.04)	(0.04)
70+ years		0.34***	0.32***
		(0.06)	(0.07)
Male		0.97	0.99
		(0.05)	(0.05)
Norwegian		0.68***	0.72***
		(0.04)	(0.04)
1 dose		1.33**	1.37**
		(0.19)	(0.20)
2 doses		1.63**	1.68*
		(0.37)	(0.39)
3 doses		1	1
		(.)	(.)
INDEX			
10-19 years			0.92
			(0.12)
20-29 years			1.26*
			(0.16)
30-39 years			1.07
			(0.13)
40-49 years			1.21
			(0.15)
50-59 years			1.18
			(0.17)
60-69 years			0.98
			(0.18)
70+ years			1.22
			(0.30)
Male			1
			(.)
Norwegian			1
			(.)
1 dose			0.85
			(0.13)
2 doses			1.00
			(0.26)
3 doses			1
			(.)
Time trends	No	Yes	Yes
Indexes	7,786	7,715	7,689
Cases	11,192	11,105	11,066

Odds ratios; Standard errors in parentheses
* $p < 0.05$, ** $p < 0.01$, *** $p < 0.001$

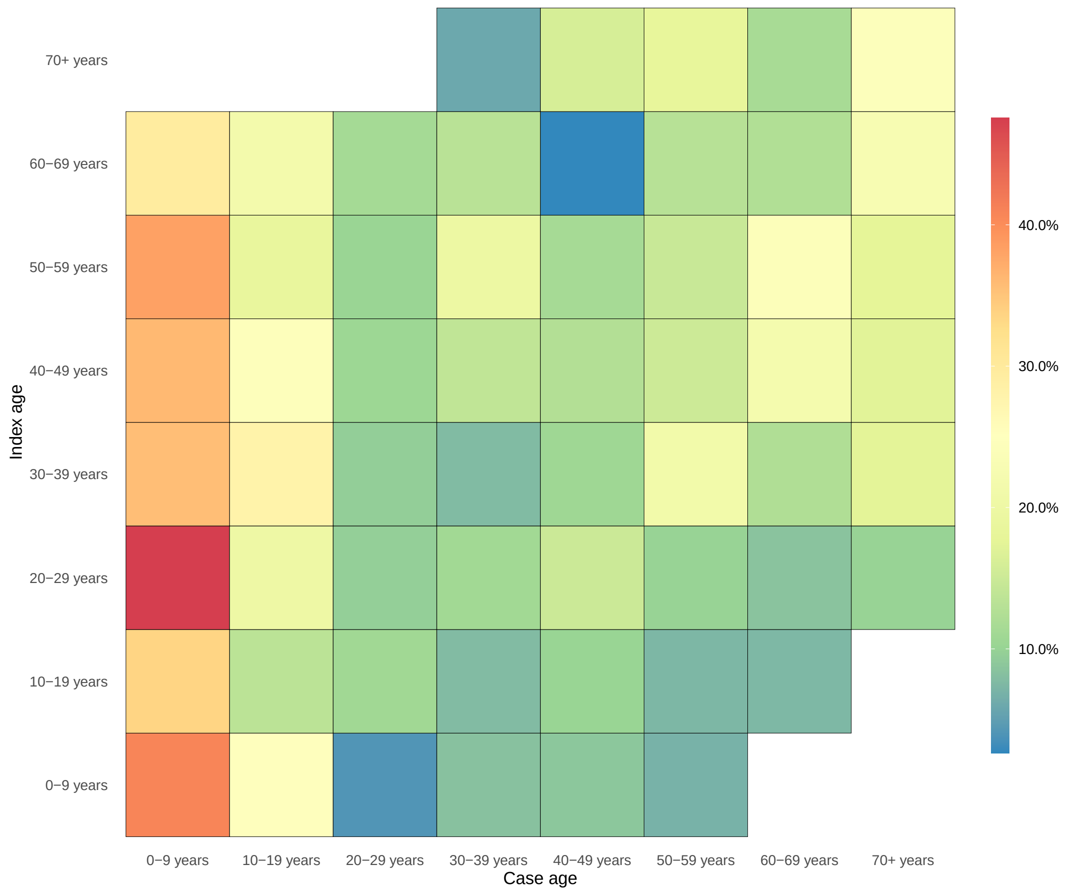

Note: Figure visualise the age combination of the assumed infector (y-axis) and the close contact (x-axis) when the close contact remained asymptomatic. E.g. 30% means that 30% of the secondary infected remained asymptomatic. Combinations with less than 10 cases were excluded (n = 34). N = 11,158.

Supplementary Fig 8: Age combinations of asymptomatic transmission.

Supplementary Table 4: Share of asymptomatic transmission by type of close contact and age.

Age group	Household member	Other
0-9 years	37%	39%
10-19 years	20%	18%
20-29 years	10%	10%
30-39 years	10%	11%
40-49 years	11%	13%
50-59 years	12%	13%
60-69 years	17%	12%
70+ years	16%	23%
Average	16%	16%
